# Severe *Falciparum* Malaria in Iran: A Very Rare Case From an Endemic Region

**DOI:** 10.5812/jjm.8752

**Published:** 2014-01-01

**Authors:** Masood Ziaee, Farshid Abedi

**Affiliations:** 1 Department of Internal Medicine, Hepatitis Research Center, Faculty of Medicine, Birjand University of Medical Sciences, Birjand, IR Iran; 2 Faculty of Medicine, Mashhad University of Medical Sciences, Mashhad, IR Iran

**Keywords:** Malaria, *Falciparum*, Immunity, Drug Therapy, Prognosis

## Abstract

**Introduction:**

Malaria is a protozoal disease, transmitted to humans by female Anopheles mosquito bite. *Plasmodium falciparum*, compared to other kinds of *Plasmodium,* causes more severe malaria and is associated with a higher mortality rate. Annually, one to three million deaths occur due to malaria, especially by *P*.* falciparum*.

**Case Presentation:**

In this report, we introduce an Iranian patient suffering from *P. falciparum*. Peripheral blood smear for malaria parasites showed severe infection of *P. falciparum*, with 75 to 85 percent of red blood cells containing one to five parasites per cell. However, the patient revealed a fast response to treatment and a good prognosis, suggesting a high level of relative immunity in the patient. To confirm this hypothesis, we conducted a comparative study by comparing the rate of clinical response to treatment as well as the level of prognosis of our patient with similar patients from different regions around the world. These included some malaria cases (caused by *P. falciparum*) chosen from endemic and nonendemic regions, such as Africa, South Europe and Canada.

**Discussion:**

The findings revealed that generally, patients from endemic regions significantly show a greater response to treatment and also a better prognosis in comparison to the patients from nonendemic regions. These differences can plausibly be attributed to a high level of relative immunity in endemic regions. Consequently, we would strongly support the hypothesis that response to treatment and prognosis of malaria is a matter of patients’ living environment circumstances. In other words, people who live in endemic regions acquire a high relative immunity leading to a greater response to treatment and a better prognosis.

## 1. Introduction

Malaria is a protozoan infection that is transmitted to humans through the bite of the Anopheles mosquito. This is the most important parasitic disease which is responsible for about one to three million deaths each year. Malaria today, as in the past, is a very big threat for tropical communities and nonendemic countries as well as travellers (1, 2). Annually, more than 2,700,000 people are at risk in malarious areas of Iran and about 3,000 malaria cases are reported (3). Annually, about 1,000 cases of malaria are reported in the United States, most of which are passengers or immigrants (4). Four species of *Plasmodium* including *P. falciparum*, *P. malariae*, *P. ovale*, and *P. vivax,* cause nearly all malaria infections in humans. However, almost all malaria-attributable mortalities are due to *P. falciparum*.

The human infection starts when the bite of an infected female Anopheles mosquito injects the *Plasmodium* sporozoites from the salivary gland into the human blood circulation. These microscopic malaria parasites rapidly travel through the bloodstream to the liver and begin the period of asexual reproduction. During this replication process, known as intrahepatic or preerythrocytic merogony step, 10,000 to more than 30,000 daughter merozoites are produced from sporozoites. Afterwards, the liver cell containing these merozoites ruptures and releases them into the bloodstream. At this stage, the infection is symptomatic. The merozoites, immediately after entering the bloodstream, invade the red blood cells (RBCs) and become trophozoite. Since *P. vivax*, *P. ovale*, and *P. malariae* have tendency to invade mature RBCs, parasitemia rarely exceeds 2%; whereas in *Falciparum* malaria, severe parasitemia occurs due to invasion to RBCs of all ages (2).

In this paper we introduce an Iranian case of severe malaria rapidly responding to treatment with intravenous quinine sulfate compared to other cases of severe malaria in the world.

## 2. Case Presentation

In January 2001, a 32-year-old man referred to Montaserieh hospital of Mashhad, Iran, with a history of fevers and vomiting 10 days after returning from Pakistan. He also complained of dark urine and hematuria. He had no history of receiving prophylactic antimalarial drugs. He had previously been hospitalized in a small hospital with a diagnosis *P. vivax* infection and had received oral chloroquine therapy. Then the patient was referred to a specialized hospital in Mashhad city due to worsening symptoms. At admission, he had a temperature of 38.5°C and his other vital signs and consciousness level were normal. On physical examination, the patient had a tender abdomen and his spleen was palpable 5 cm below the left costal margin.

**Figure 1.**
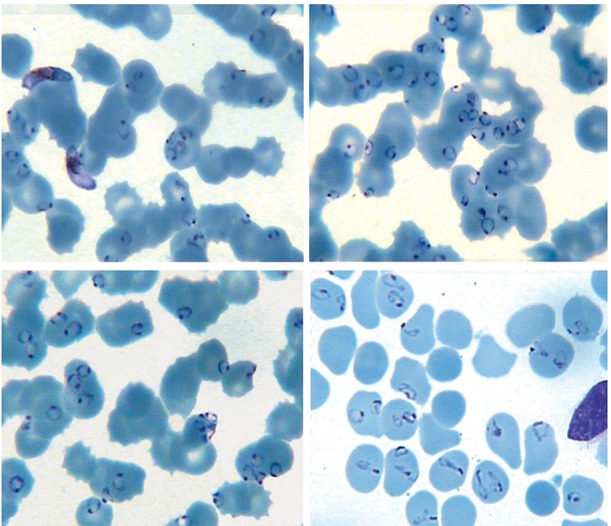
Peripheral Blood Smear of a 32-Year-Old Iranian Patient With a Severe Infection of *P. falciparum* Among RBCs, 75 to 85% contained one to five parasites per cell (Giemsa stain, X1000)

In laboratory studies, hemoglobin was 11.5 g/dL, hematocrit 34%, white blood cell count 16,000/mm^3 ^, and platelet count was 45,000/mm ^3 ^. His creatinine level was 1.9 mg/dL. Serum was assayed for total bilirubin (Bil), lactate dehydrogenase (LDH), aspartate aminotransferase (AST), and alanine aminotransferase (ALT), using commercially available kits. The serum level of Bil was 2.3 mg/dL, LDH 1680 U/L (normal level: 80 - 100 U/L), AST 106 U/L, and ALT 29 U/L. Peripheral blood smear examination showed severe infection with *P. falciparum *and 75 to 85% of RBCs contained one to five parasites per cell (Figure 1). 

Patient probably had a mixed infection with *P. vivax* and *P. falciparum* that the *P. vivax* infection had been treated with oral chloroquine, but chloroquine-resistant *P. falciparum* remained and parasitemia had increased.

The patient was treated with intravenous quinine sulfate and oral primaquine. Then, he received primaquine (30 mg a day for 14 days) after the symptoms improvement for treating the probable mixed malaria infection. The improvement was considerable, so that parasitemia decreased by 40% after 24 hours and 1% after 4 days of posttreatment. During the treatment process, the patient developed dark urine and elevated creatine phosphokinase; thus, progression to acute renal failure was considered for him. Acute respiratory distress syndrome (ARDS) was exhibited in bilateral pulmonary infiltrates on the chest X-ray; also, he presented confusion due to cerebral malaria or superimposed a Gram-negative infection, so ceftriaxone was prescribed. However, patient was discharged after 10 days with no requirement for intubation, hemodialysis, or ICU admission. He had a good general condition at the last follow-up visit.

## 3. Discussion

Severe *falciparum* malaria is a medical emergency that requires intensive care. Parenteral quinine or quinidine should be administered if any doubt exists about drug resistance. In very ill patients, blood exchange transfusion should be considered and it is indicated in high parasitemia (more than 30%) (5). The patient presented in this paper was important in two aspects: one was because of high parasitemia which was certainly a very rare condition and the only similar reported case in the world with high parasitemia as our patients was a Swiss case (with a parasitemia of 70 - 80%), and second, the rapid response to treatment with intravenous quinine without the necessity of blood exchange or other supportive care proceedings (such as hemodialysis, ventilator, or ICU admission) and improvement after 10 days hospitalization.

**Figure 2.**
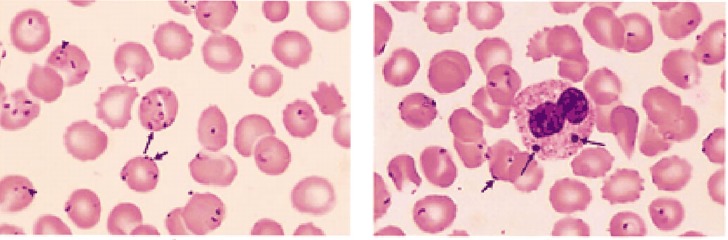
Peripheral Blood Smear in a 34-Year-old Swiss Patients With Severe Malaria Among RBCs, 70 to 80% contained up to five *P. falciparum* cells (7).

Outcomes of sever malaria differ in immune and nonimmune patients (6). We found some reports of immune and nonimmune patients with severe malaria. The first case was a 34-year-old Swiss patient with a history of travel to Madagascar presenting severe *P. falciparum *malaria in which 70 - 80% of RBCs in the peripheral blood smear were infected (Figure 2) (7). The treatment course took about 3 months and he was hospitalized during this process. In addition to administration of intravenous quinine sulfate, he underwent blood exchange transfusion as well as laparotomy and splenectomy due to splenic rupture. Also, he was placed on mechanical ventilation, and underwent hemodialysis. Although the rate of parasitemia was similar to that of our patient, response to the treatment was completely different (7). 

The other case was a 54-year-old patient, progressed to severe malaria (*P. falciparum*) after returning from Kenya with a parasitemia rate of 10.4% (8). Treatment with intravenous quinine and doxycycline were initiated for him. Despite the elimination of parasites from the blood, patient died from pulmonary edema and heart failure due to water and electrolyte abnormalities. Although the percentage of parasitemia was lower and even the treatment contained broad-spectrum drugs, he was a nonimmune case from a nonendemic region and so had a poorer prognosis than the two previously mentioned cases.

The other patient was a 17-year-old UK-resident woman who was born in Nigeria. She presented a maximum parasitemia of 30% (*P. falciparum*) after returning from her native country. Although she was admitted to ICU and undergone ventilation and hemodialysis, she was discharged with good general conditions and no need for blood exchange transfusion on the day 10 after the admission. In this case, although the disease progress was associated with more complications than ours, no need for blood exchange as well as the short length of hospital stay (10 days) indicated the relative immunity of patient which was due to her birthplace and history of living in an endemic region (9).

Other case was a 29-year-old Canadian man who had non-specific symptoms of malaria after returning from Zambia. The peripheral blood smear was negative for malarial parasite. The patient’s consciousness was impaired 48 hours later and he died in the next few hours. *P. falciparum* was reported as the cause of death in autopsy. Although this patient received no medical treatment, the rapid and fatal course of malaria infection even with negative parasitemia in a nonimmune patient living in a nonendemic region, draws our attention to the importance of the relative immunity of people living in endemic regions (10).

Finally, in another article, four patients with *P. falciparum* were introduced (two 50-year-old Canadian patients, male and female, with parasitemia of 1%, a 28-year-old Norwegian patient with parasitemia of 2%, and a 64-year-old Indian patient with parasitemia of 6%). The Indian patient, compared to the three others (who lived in nonendemic regions) despite a higher rate of parasitemia, had lower symptoms and a faster response to treatment (11). Presumably, the theory of a relative immunity existence among people living in the malaria endemic regions, leads to a faster response to treatments and better prognosis. This is quite reasonable considering the collected data and cases from around the world. Therefore, the indications of blood exchange, ICU admission, laparotomy, and splenectomy, as well as the disease prognosis in severe malaria, can be different based on a person’s location and immunity.
